# Supplementary material for: Engineering of a mammalian VMAT2 for cryo-EM analysis results in non-canonical protein folding
Source: Nat Commun. 2024 Aug 2;15:6511. doi: 10.1038/s41467-024-50934-5 (PMC11297040; doi:10.1038/s41467-024-50934-5)
Supplement: Supplementary file 1 — Supplementary Information [file 41467_2024_50934_MOESM1_ESM.pdf]

Supplementary Information for

Engineering of a mammalian VMAT2 for cryo-EM analysis results in non-canonical protein folding

Ying Lyu[1‡], Chunting Fu[1‡], Haiyun Ma[2‡], Zhaoming Su[2*], Ziyi Sun[1*], Xiaoming Zhou[1*]

[1]Department of Integrated Traditional Chinese and Western Medicine, State Key Laboratory of Biotherapy, West China Hospital, Sichuan University, Chengdu, Sichuan 610041, China

[2]State Key Laboratory of Biotherapy, Department of Geriatrics and National Clinical Research Center for Geriatrics, West China Hospital, Sichuan University, Chengdu, Sichuan 610041, China

[‡]These authors contributed equally to this work.

*To whom correspondence should be addressed:

Zhaoming Su, PhD, 17 Renmin Road South 3rd Section, Sichuan University, Chengdu, Sichuan 610041, China; Email: zsu@wchscu.cn

Ziyi Sun, PhD, 17 Renmin Road South 3rd Section, Sichuan University, Chengdu, Sichuan 610041, China; Email: ziyi.sun@scu.edu.cn

Xiaoming Zhou, PhD, 17 Renmin Road South 3rd Section, Sichuan University, Chengdu, Sichuan 610041, China; Email: x.zhou@scu.edu.cn

Supplementary figures and tables

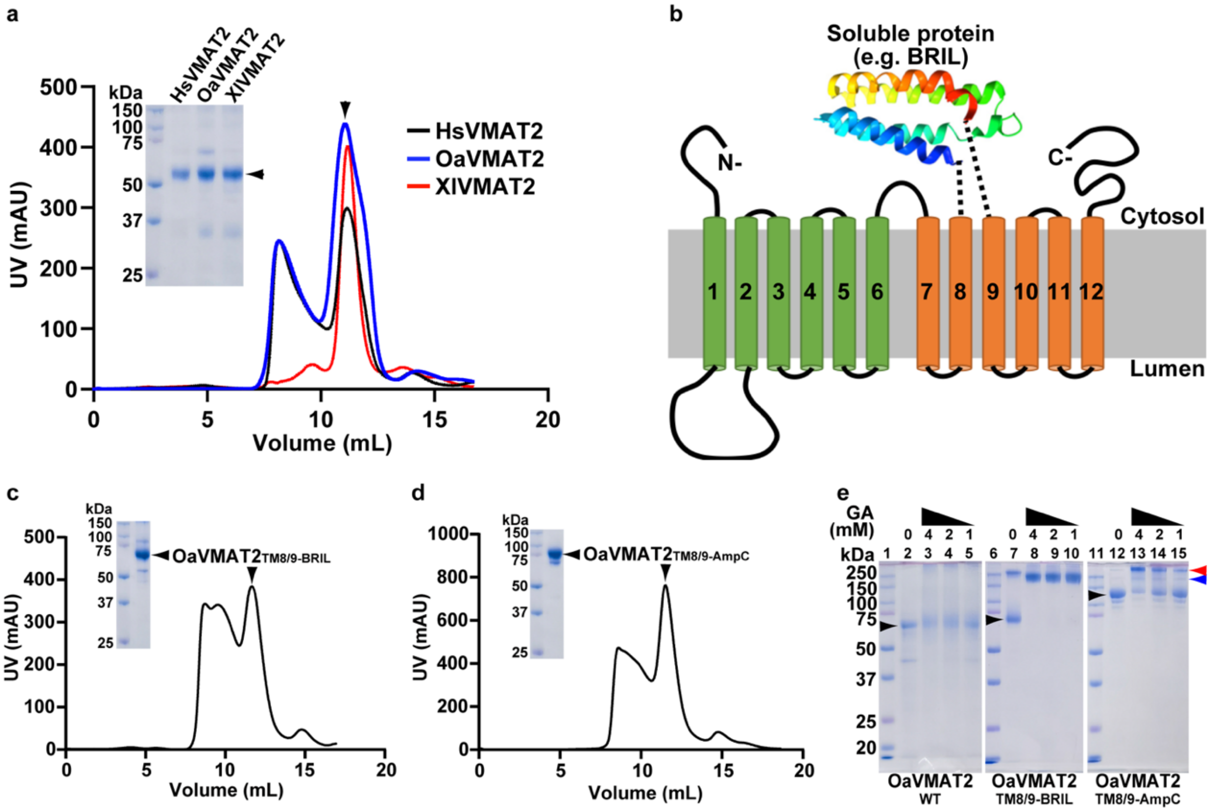

Supplementary Figure 1. Engineering and purification of VMAT2s. (a) Gel-filtration profiles of wild-type HsVMAT2, OaVMAT2 and XlVMAT2 in DDM. Source data are provided as a Source Data file. Arrowheads indicate VMAT2 peaks and VMAT2 bands (inset). (b) A schematic showing VMAT2 engineering by replacing inter-helix loops with soluble proteins (e.g. BRIL). VMAT2 N- and C-domains are colored in green and orange, respectively. Twelve TM helices of VMAT2 are labeled with numbers throughout the manuscript, and the membrane is indicated by a grey rectangle. (c)-(d) Gel-filtration profiles of OaVMAT2$_{TM8/9\text{-}BRIL}$ (panel c) and OaVMAT2$_{TM8/9\text{-}AmpC}$ (panel d) in DDM. Source data are provided as a Source Data file. Arrowheads indicate OaVMAT2 peaks and OaVMAT2 bands (inset). (e) Chemical crosslinking of OaVMAT2 WT and variants by various concentrations of glutaraldehyde (GA) as indicated. Chemical crosslinking and SDS-PAGE experiments were repeated three times with similar results. Lanes 1, 6 and 11 show protein markers with molecular weights indicated on the left of the gel. The black, blue and red arrowheads indicate monomer, dimer and high-molecular-weight aggregate bands, respectively. In panels a, c and d, the unit for the y-axis (ultraviolet 280 absorbance, UV) is milli-Arbitrary Unit (mAU) as used by ÄKTA pure purifiers (Cytiva).

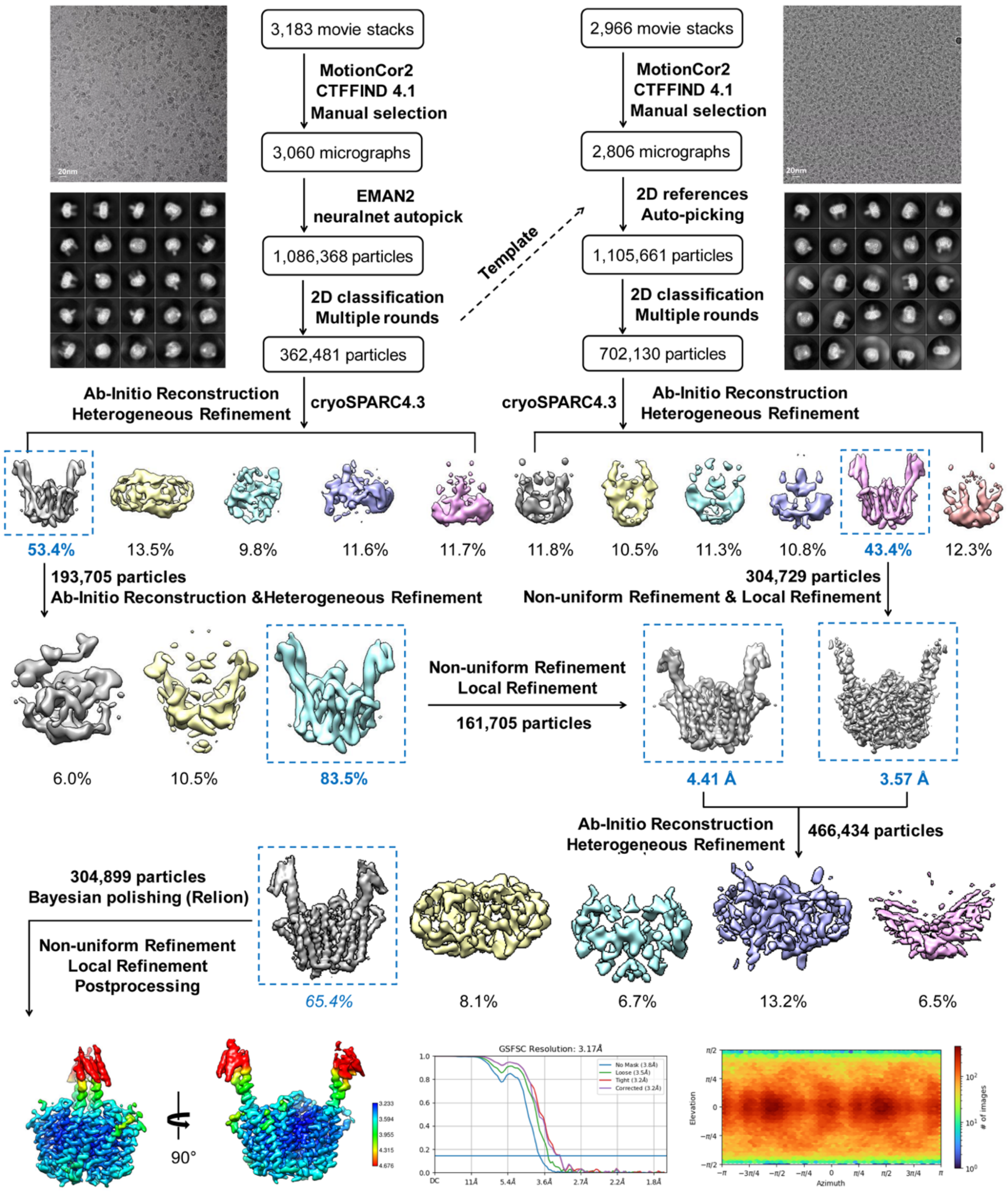

Supplementary Figure 2. Workflow of cryo-EM data processing of OaVMAT2$_{TM8/9\text{-}BRIL}$ datasets. Referring to Methods section for details.

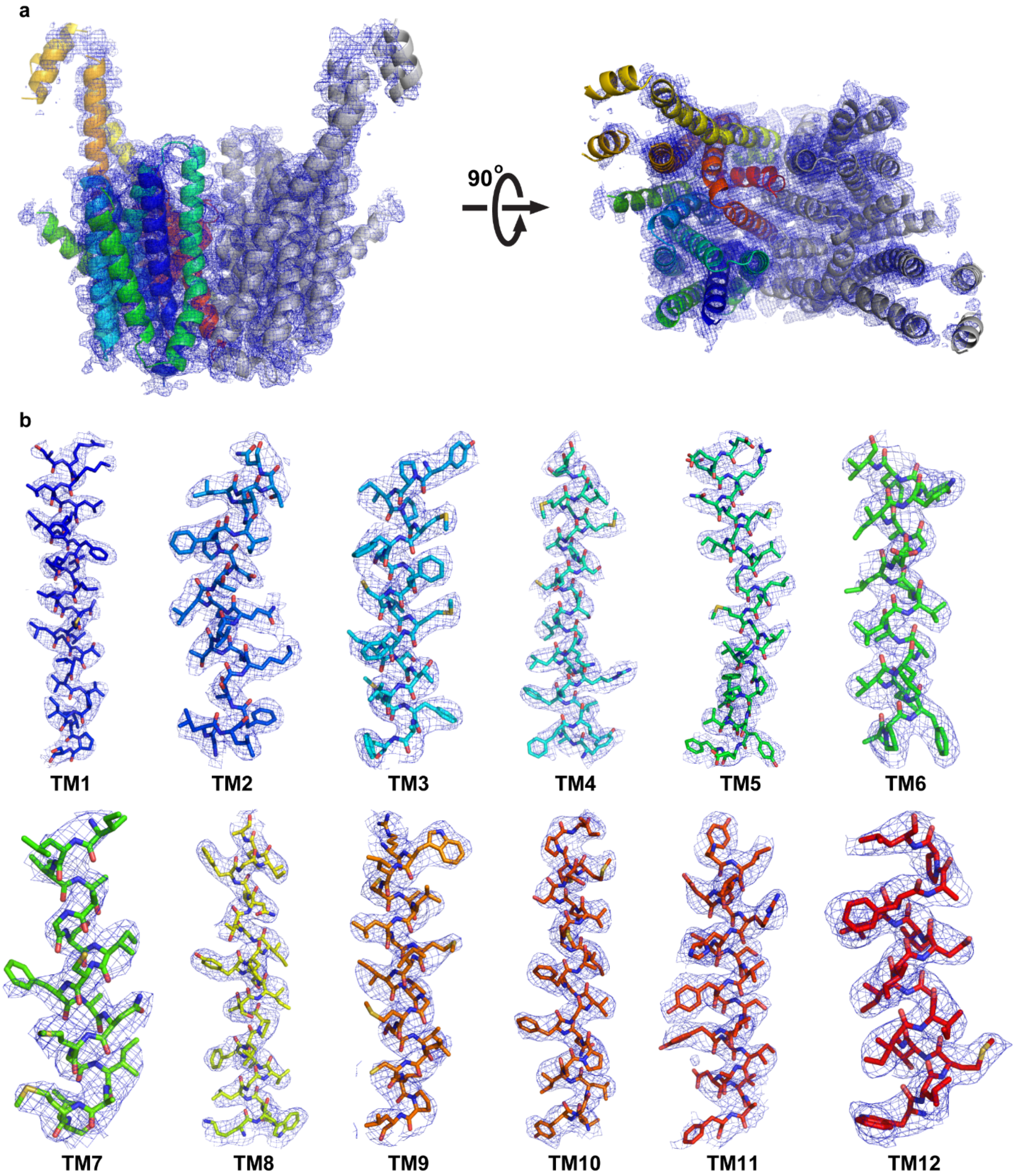

Supplementary Figure 3. OaVMAT2$_{TM8/9\text{-}BRIL}$ model building into cryo-EM density map. (a) OaVMAT2$_{TM8/9\text{-}BRIL}$ dimer built into OaVMAT2$_{TM8/9\text{-}BRIL}$ dimer density. Protomer A is displayed in spectrum color, while protomer B in grey. Left, viewed parallel to the membrane. Right, viewed perpendicular to the membrane from the cytosolic side. (b) Fitting of each TM helix of OaVMAT2$_{TM8/9\text{-}BRIL}$ protomer A in its density. Blue mesh indicates cryo-EM density map contoured at 5.0 σ level.

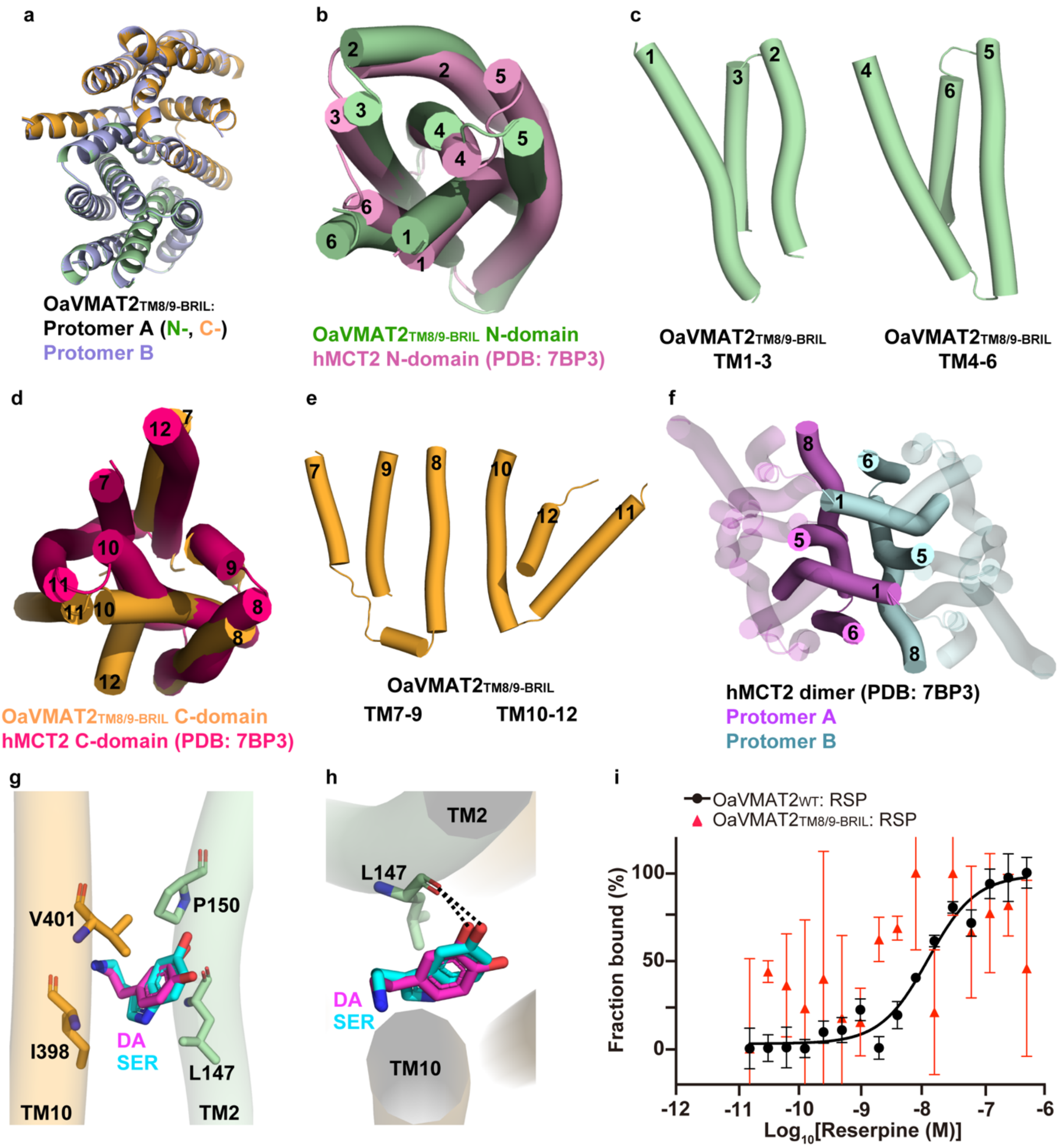

Supplementary Figure 4. Analysis of the OaVMAT2$_{TM8/9\text{-}BRIL}$ structure. (a) Superposition of OaVMAT2$_{TM8/9\text{-}BRIL}$ protomer B (in light blue) onto protomer A (N-domain in green and C-domain in orange). (b) Superposition of OaVMAT2$_{TM8/9\text{-}BRIL}$ N-domain (in green) onto hMCT2 N-domain (PDB: 7BP3, in pink). (c) Comparison between OaVMAT2$_{TM8/9\text{-}BRIL}$ TM1-3 (left) and TM4-6 (right). (d) Superposition of OaVMAT2$_{TM8/9\text{-}BRIL}$ C-domain (in orange) onto hMCT2 C-domain (PDB: 7BP3, in dark pink). (e) Comparison between OaVMAT2$_{TM8/9\text{-}BRIL}$ TM7-9 (left) and TM10-12 (right). (f) The dimer interface of the hMCT2 dimer (PDB: 7BP3) with protomer A in purple and protomer B in light cyan. Interface-forming TMs are labeled with numbers. (g) Potential hydrophobic interactions between docked DA (magenta sticks) or SER (cyan

sticks) and $OaVMAT2_{TM8/9\text{-}BRIL}$ (orange and green cartoons). Participating residues are shown as sticks. (h) Potential hydrogen bonds between docked DA (magenta sticks) or SER (cyan sticks) and main-chain carbonyl oxygen of L147 (green sticks) of TM2 are indicated by black dashed lines. (i) MST fitting curves of RSP binding to $OaVMAT2_{WT}$ and $OaVMAT2_{TM8/9\text{-}BRIL}$, *N*=3 repeats with biologically independent protein samples. Data are presented as mean $\pm$ SEM. Source data are provided as a Source Data file.

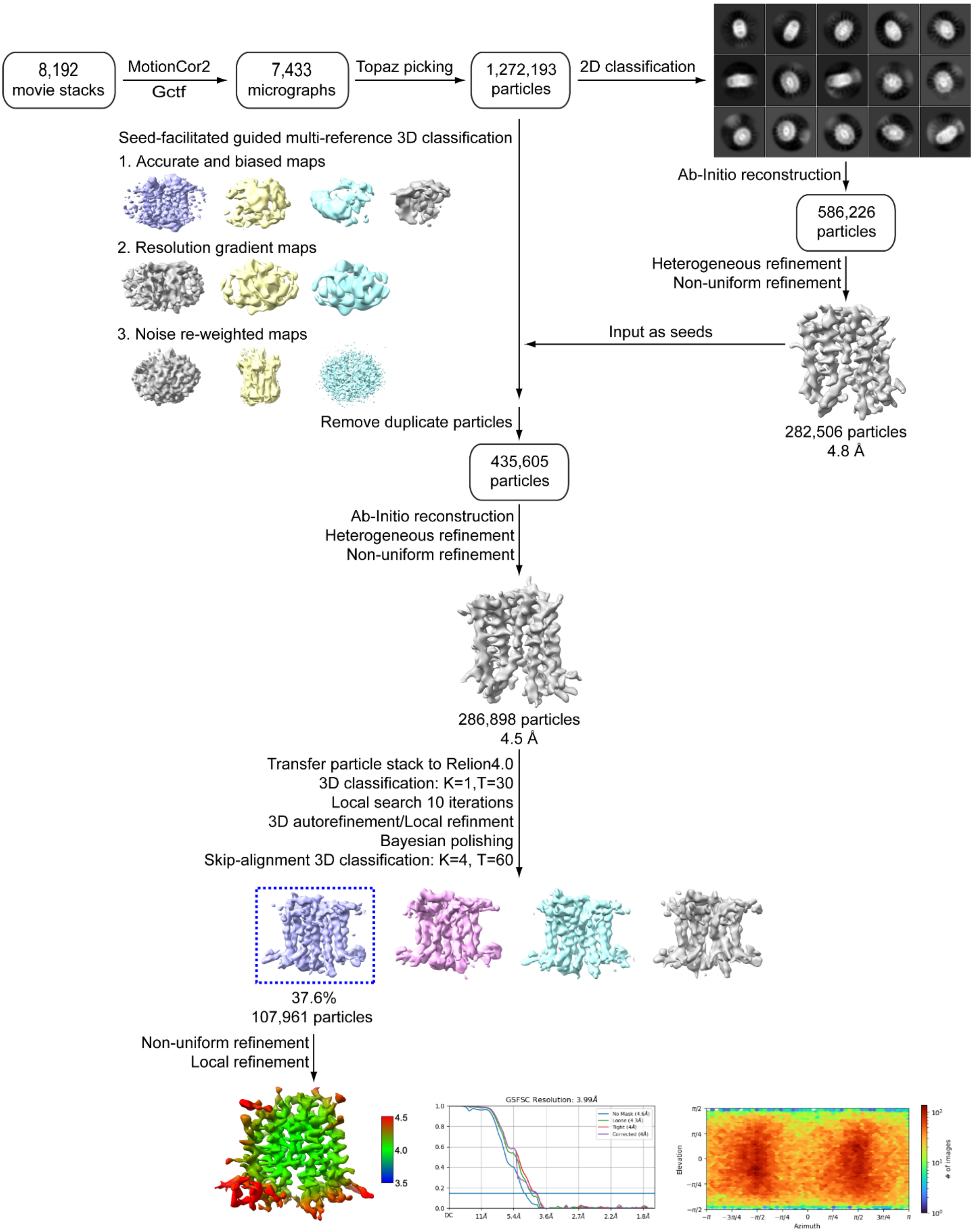

Supplementary Figure 5. Workflow of cryo-EM data processing of XlVMAT2$_{WT}$ dataset.

Referring to Methods section for details.

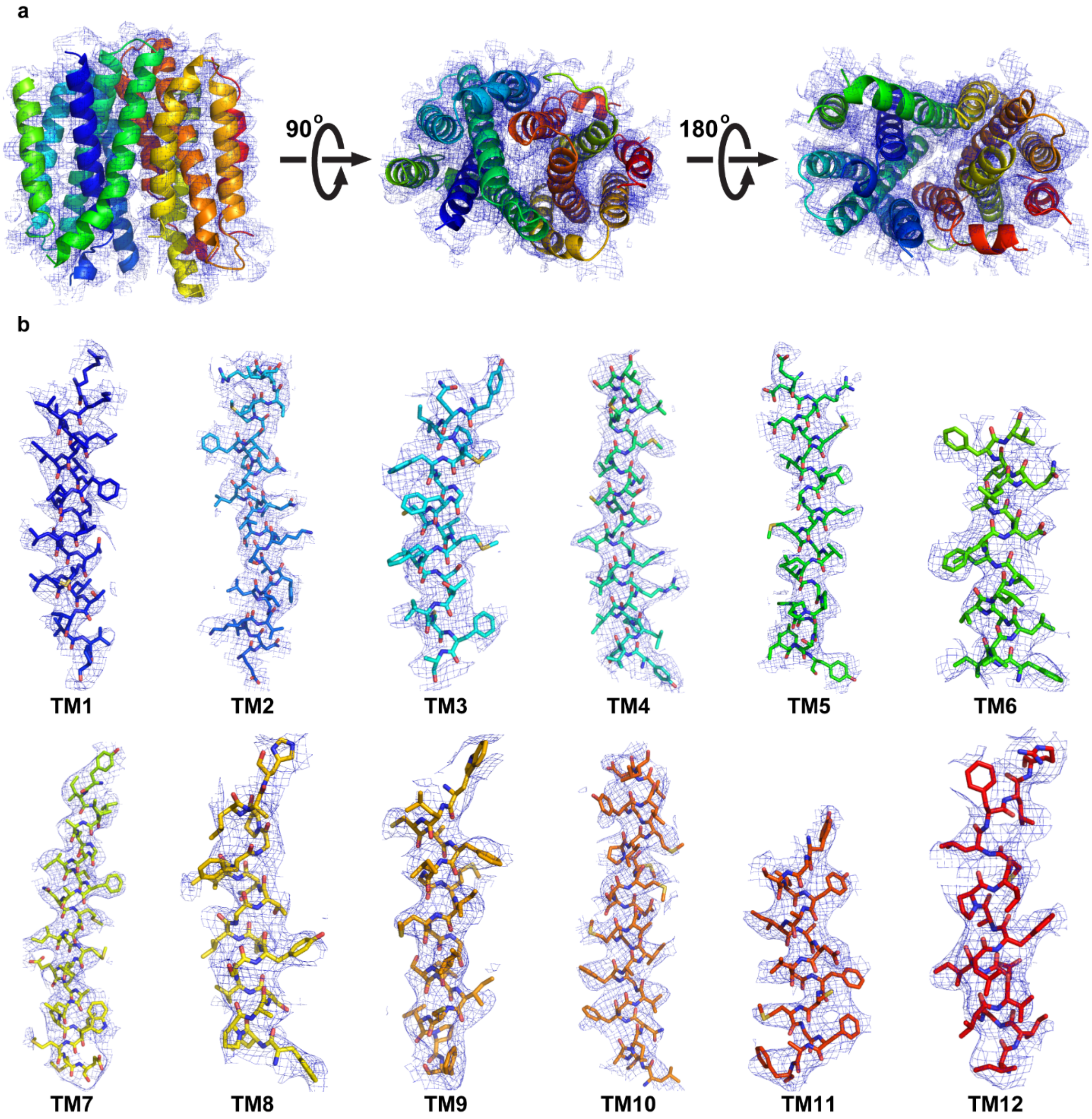

Supplementary Figure 6. XlVMAT2$_{WT}$ model building into cryo-EM density map. (a) XlVMAT2$_{WT}$ monomer (in spectrum color) built into XlVMAT2$_{WT}$ monomer density. Left, viewed parallel to the membrane. Middle, viewed perpendicular to the membrane from the cytosolic side. Right, viewed perpendicular to the membrane from the luminal side. (b) Fitting of each TM helix of XlVMAT2$_{WT}$ in its density. Blue mesh indicates cryo-EM density map contoured at 5.0 σ level.

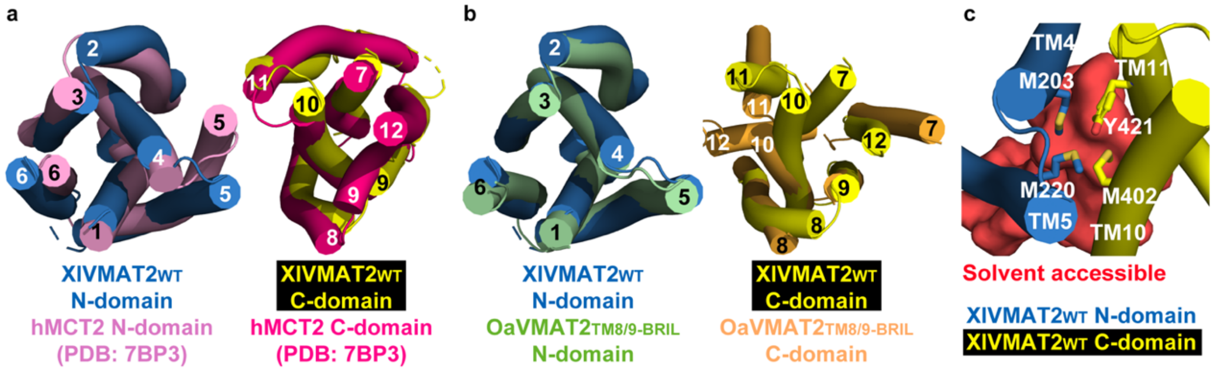

Supplementary Figure 7. Analysis of the XlVMAT2$_{WT}$ structure. (a) Superposition of XlVMAT2$_{WT}$ monomer and hMCT2 protomer (PDB: 7BP3). Left, structural alignment of N-domains (XlVMAT2$_{WT}$ in blue and hMCT2 in pink). Right, structural alignment of C-domains (XlVMAT2$_{WT}$ in yellow and hMCT2 in dark pink). (b) Superposition of XlVMAT2$_{WT}$ monomer and OaVMAT2$_{TM8/9\text{-}BRIL}$ protomer. Left, structural alignment of N-domains (XlVMAT2$_{WT}$ in blue and OaVMAT2$_{TM8/9\text{-}BRIL}$ in green). Right, structural alignment of C-domains (XlVMAT2$_{WT}$ in yellow and OaVMAT2$_{TM8/9\text{-}BRIL}$ in orange). (c) The cytosolic gate of XlVMAT2$_{WT}$ formed by M203, M220, M402 and Y421 (shown as sticks), viewed perpendicular to the membrane from the cytosolic side. The solvent-accessible space is displayed as red surface.

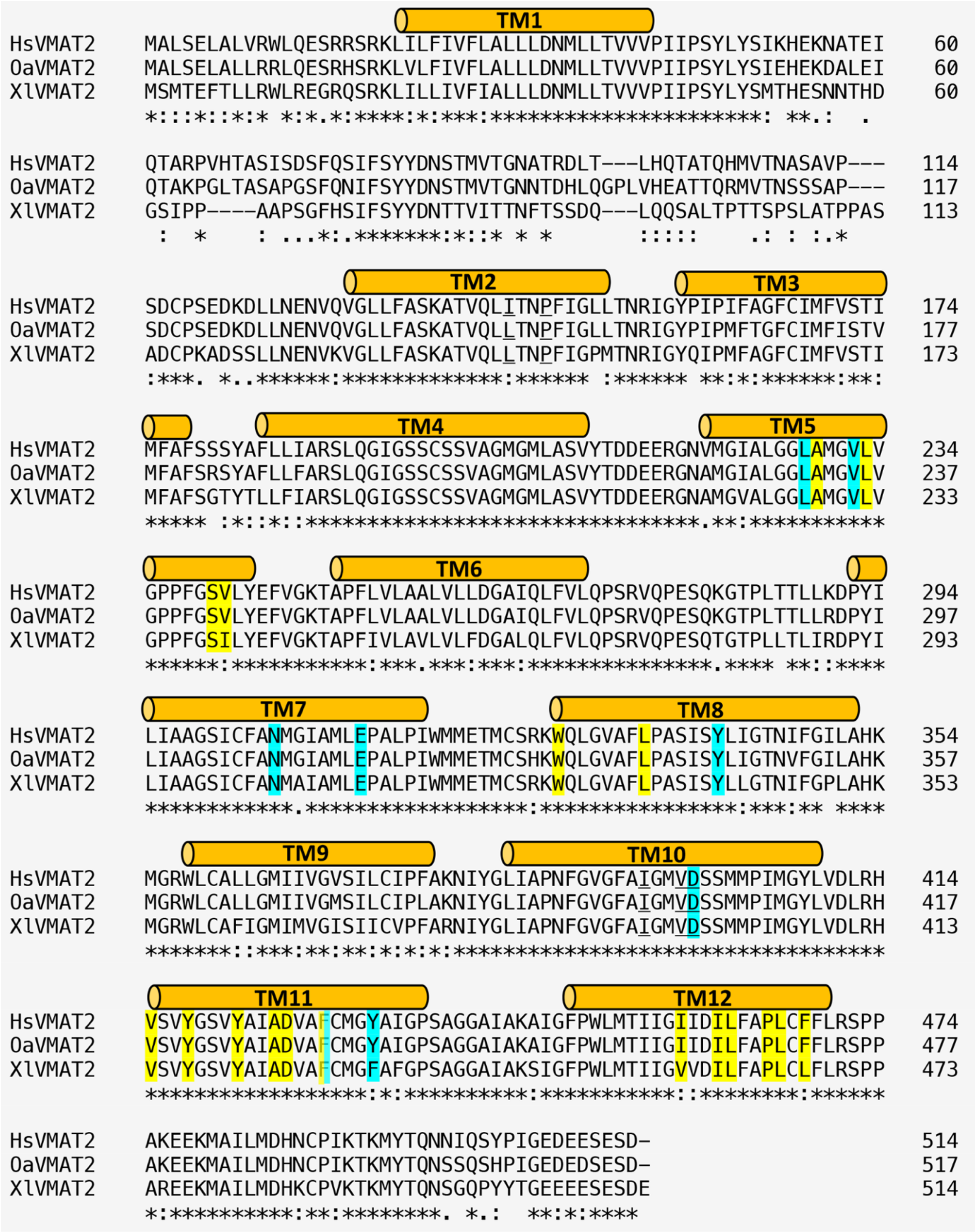

Supplementary Figure 8. Sequence alignment of VMAT2s by ClustalW[1,2]. TM helices are indicated by cylinders and labeled from TM1 to TM12. Residues forming the dimer interface in OaVMAT2$_{TM8/9\text{-}BRIL}$ are highlighted in yellow. Residues interacting with docked SER in XlVMAT2$_{WT}$ are highlighted in cyan. Residues interacting with docked DA/SER in OaVMAT2$_{TM8/9\text{-}BRIL}$ are underscored. Asterisks (*) indicate identical residues. Colons (:)

indicate strong similarities. Periods (.) indicate weak similarities. Hs, *Homo sapiens*; Oa, *Ovis aries*; Xl, *Xenopus laevis*.

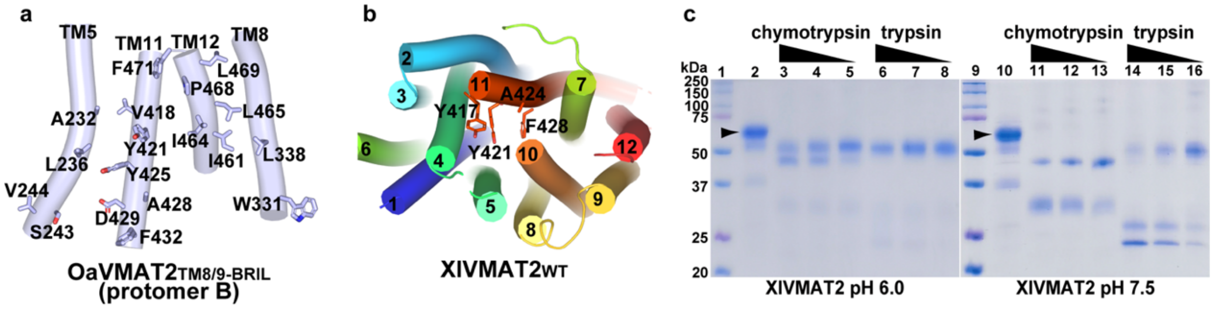

Supplementary Figure 9. The dimer interface of OaVMAT2$_{\mathrm{TM8/9\text{-}BRIL}}$. (a) One protomer of the OaVMAT2$_{\mathrm{TM8/9\text{-}BRIL}}$ dimer interface (TM5, TM8, TM11 and TM12) is shown as cylinders. Residues participating in formation of the dimer interface are displayed in sticks. (b) The XlVMAT2$_{\mathrm{WT}}$ structure viewed perpendicular to the membrane from the cytosolic side. Several residues on TM11 in XlVMAT2 (Y417, Y421, A424 and F428 equivalent to Y421, Y425, A428 and F432 in OaVMAT2) are shown as sticks. (c) Enzymatic (trypsin and chymotrypsin) digestion patterns of purified XlVMAT2 proteins at pHs 6.0 and 7.5. Enzymatic digestion and SDS-PAGE experiments were repeated three times with similar results. For each digestion series, the three enzyme ratios (from high to low) are 10:1, 20:1 and 50:1 (w/w, XlVMAT2:enzyme). Lanes 1 and 9 show protein markers with molecular weights indicated on the left of the gel. The black arrowhead indicates purified XlVMAT2 before digestion.

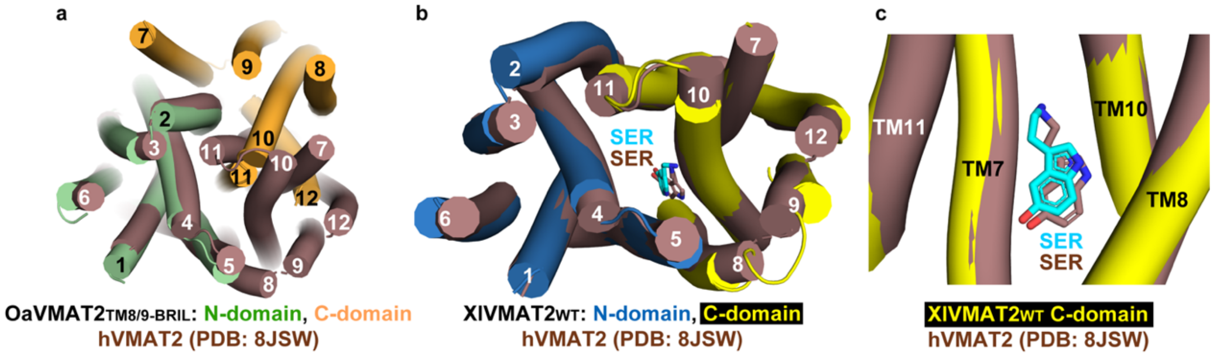

Supplementary Figure 10. Comparison of VMAT2 structures. (a) Superposition of OaVMAT2$_{TM8/9\text{-}BRIL}$ protomer (N-domain in green and C-domain in orange) onto a hVMAT2 structure (in brown, PDB: 8JSW) by aligning their N-domains. Viewed perpendicular to the membrane from the cytosolic side. (b) Superposition of XlVMAT2$_{WT}$ (N-domain in blue and C-domain in yellow) onto a hVMAT2-SER structure (in brown, PDB: 8JSW). Viewed perpendicular to the membrane from the cytosolic side. The docked SER (cyan sticks) in XlVMAT2$_{WT}$ and the bound SER (brown sticks) in hVMAT2 are shown. (c) A close-up view of panel b from a different angle with the focus of SER.

Supplementary Table 1. VMAT2 engineering by replacing various inter-helix loops with soluble proteins.

VMAT2 name	Loop identifier	Loop replaced (residue #)	Soluble protein insert	Linker sequence (N linker-C linker)	Fusion protein expression	Gel-filtration profile	Fusion protein Name
	TM1/2	51-125	BRIL	-	Weak	Aggregation	-
HsVMAT2	TM6/7	279-280	BRIL	-	No	-	-
	TM6/7	277-285	AmpC	-	No	-	-
	TM1/2	51-125	AmpC	-	Weak	Aggregation	-
	TM1/2	46-125	AmpC	-	Weak	Aggregation	-
	TM4/5	216-224	AmpC	-	No	-	-
	TM6/7	280-287	AmpC	-	Weak	Aggregation	-
	TM6/7	274-293	AmpC	-	Weak	Aggregation	-
	**TM8/9**	**356-359**	**AmpC**	**-**	**OK**	**OK**	**OaVMAT2$_{TM8/9\text{-}AmpC}$**
OaVMAT2	TM8/9	355-359	AmpC	-	Weak	Aggregation	-
	TM8/9	355-360	AmpC	RRQL-ERARS	No	-	-
	TM8/9	355-360	AmpC	ARRQL-ERARS	Weak	Aggregation	-
	TM8/9	355-360	AmpC	RRQL-ERARSTL	No	-	-
	TM8/9	355-360	BRIL	RRQL-ERARS	Weak	Aggregation	-
	TM8/9	355-360	BRIL	ARRQL-ERARS	No	-	-
	**TM8/9**	**355-360**	**BRIL**	**RRQL-ERARSTL**	**OK**	**OK**	**OaVMAT2$_{TM8/9\text{-}BRIL}$**
	TM6/7	274-284	BRIL	-	No	-	-
XlVMAT2	TM6/7	270-289	BRIL	-	Weak	Aggregation	-
	TM6/7	270-289	AmpC	-	No	-	-

Supplementary Table 2. Affinities (K_d) of substrate/inhibitor to VMAT2 determined by MST.

Name	Substrate /Inhibitor	Variant	Repeat 1 (μM*)	Repeat 2 (μM)	Repeat 3 (μM)	Mean (μM)	SEM (μM)	*P*
Oa VMAT2	DA	WT	1.97	0.34	0.30	0.87	0.55	-
		TM8/9-BRIL	1.06	0.72	0.25	0.68	0.23	0.76
		TM8/9-BRIL D402A	13.4	12.6	7.5	11.2	1.8	0.0049
	SER	WT	0.28	0.27	0.09	0.21	0.06	-
		TM8/9-BRIL	0.48	0.37	0.14	0.33	0.10	0.38
		TM8/9-BRIL D402A	21.4	23.9	14.8	20.0	2.7	0.0019
	RSP	WT	27.3 nM	11.2 nM	9.6 nM	16.0 nM	5.7 nM	-
		TM8/9-BRIL	-	-	-	-	-	-
Xl VMAT2	DA	WT	0.46	0.76	2.16	1.13	0.52	-
	SER	WT	0.27	0.15	0.86	0.43	0.22	-
	RSP	WT	6.6 nM	9.9 nM	34.4 nM	17.0 nM	8.8 nM	-
Hs VMAT2	SER	WT	0.38	0.42	0.56	0.45	0.05	-
		L228A	4.16	2.25	3.04	3.15	0.55	0.0084
		V232A	0.96	6.51	4.09	3.85	1.61	0.10
		E312A	-	-	-	-	-	-
		Y341A	0.81	1.19	0.44	0.81	0.22	0.18
		D399A	25.2	14.9	26.3	22.1	3.6	0.0039
		Y433A	3.99	6.12	1.61	3.91	1.30	0.057

MST measurements were repeated with N=3 biologically independent protein samples and source data of K_d values are shown. Two-tailed Student's t-test was performed between WT and other variants of each VMAT2 for each substrate/inhibitor, except that the "TM8/9-BRIL D402A" group was compared to the "TM8/9-BRIL" group, and the *P* values are shown. *All K_d values are shown in μM unless otherwise indicated.

Supplementary Table 3. FFN200 uptake by HEK293T cells expressing various VMAT2 variants.

Name	Variant	Repeat 1 (fluorescence, AU*)	Repeat 2 (fluorescence, AU)	Repeat 3 (fluorescence, AU)	Normalized mean# (%)	Normalized SEM# (%)	*P*
Oa VMAT2	WT	35.0	29.9	36.6	100.0	6.0	-
	TM8/9-BRIL	23.5	20.6	18.6	61.8	4.2	-
	TM8/9-BRIL D402A	9.9	14.6	12.4	36.4	4.0	0.012
Xl VMAT2	WT	29.8	24.6	15.7	69.1	12.2	-
Hs VMAT2	WT	60.6	50.9	61.9	170.8	10.3	-
	E312A	10.0	8.4	13.1	31.0	4.1	0.00022
	D399A	14.0	17.0	20.3	50.5	5.4	0.00049

FFN200 uptake experiments were repeated with *N*=3 biologically independent cell samples and source data of accumulated fluorescence are shown. Two-tailed Student's t-test was performed between the "TM8/9-BRIL D402A" group and the "TM8/9-BRIL" group for OaVMAT2, and between WT and other variants for HsVMAT2, and the *P* values are shown. *Fluorescence unit is Arbitrary Unit (AU) for Duetta fluorescence spectrometer (HORIBA Scientific). #Fluorescent signals were normalized in a 0-100% scale, using the signal from cells expressing wild-type OaVMAT2 as 100%.

Supplementary References

1. Thompson, J.D., Higgins, D.G. & Gibson, T.J. CLUSTAL W: improving the sensitivity of progressive multiple sequence alignment through sequence weighting, position-specific gap penalties and weight matrix choice. *Nucleic Acids Res* **22**, 4673-80 (1994).
2. Combet, C., Blanchet, C., Geourjon, C. & Deleage, G. NPS@: network protein sequence analysis. *Trends Biochem Sci* **25**, 147-50 (2000).

Uncropped gels

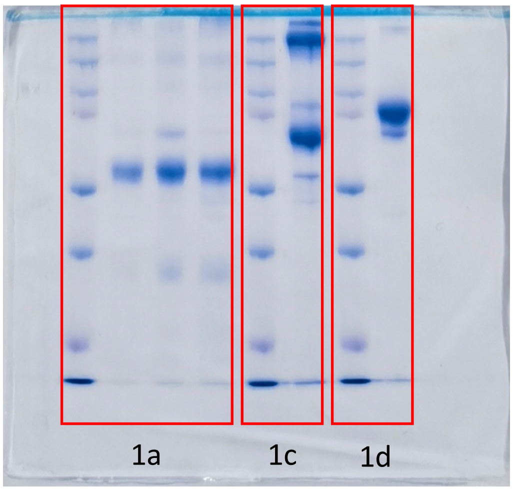

Supplementary Figure 1a, 1c and 1d.

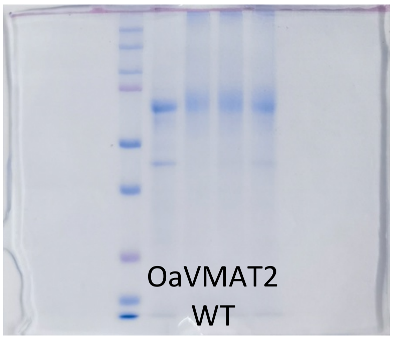

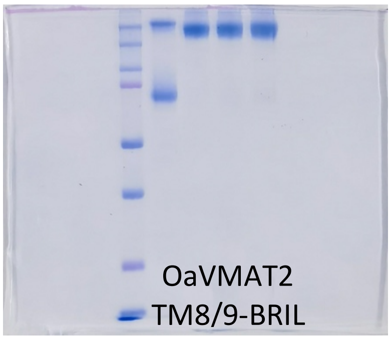

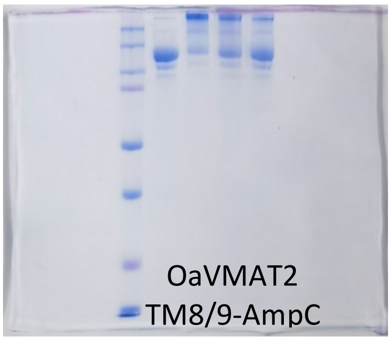

Supplementary Figure 1e.

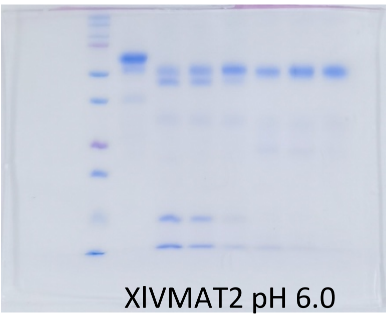

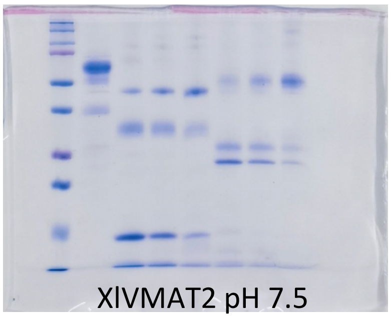

Supplementary Figure 9c.
