# Supplementary material for: Engineering of a mammalian VMAT2 for cryo-EM analysis results in non-canonical protein folding
Source: Nat Commun. 2024 Aug 2;15:6511. doi: 10.1038/s41467-024-50934-5 (PMC11297040; doi:10.1038/s41467-024-50934-5)
Supplement: Supplementary file 3 — Reporting Summary [file 41467_2024_50934_MOESM3_ESM.pdf]

nature portfolio

Corresponding author(s): Zhaoming Su, Ziyi Sun, Xiaoming Zhou

Last updated by author(s): Jun 24, 2024

Reporting Summary

Nature Portfolio wishes to improve the reproducibility of the work that we publish. This form provides structure for consistency and transparency in reporting. For further information on Nature Portfolio policies, see our Editorial Policies and the Editorial Policy Checklist.

Statistics

For all statistical analyses, confirm that the following items are present in the figure legend, table legend, main text, or Methods section.

n/a	Confirmed	
☐	☒	The exact sample size (n) for each experimental group/condition, given as a discrete number and unit of measurement
☐	☒	A statement on whether measurements were taken from distinct samples or whether the same sample was measured repeatedly
☐	☒	The statistical test(s) used AND whether they are one- or two-sided<br>*Only common tests should be described solely by name; describe more complex techniques in the Methods section.*
☒	☐	A description of all covariates tested
☒	☐	A description of any assumptions or corrections, such as tests of normality and adjustment for multiple comparisons
☐	☒	A full description of the statistical parameters including central tendency (e.g. means) or other basic estimates (e.g. regression coefficient) AND variation (e.g. standard deviation) or associated estimates of uncertainty (e.g. confidence intervals)
☐	☒	For null hypothesis testing, the test statistic (e.g. F, t, r) with confidence intervals, effect sizes, degrees of freedom and P value noted<br>*Give P values as exact values whenever suitable.*
☒	☐	For Bayesian analysis, information on the choice of priors and Markov chain Monte Carlo settings
☒	☐	For hierarchical and complex designs, identification of the appropriate level for tests and full reporting of outcomes
☒	☐	Estimates of effect sizes (e.g. Cohen's d, Pearson's r), indicating how they were calculated

Our web collection on statistics for biologists contains articles on many of the points above.

Software and code

Policy information about availability of computer code

Data collection	For cryo-EM data collection: EPU software Version 2.9.0.1519REL.<br>For MST measurement: MO.Control v1.6.1.
Data analysis	For cryo-EM data processing: EMAN v2.2, Relion v4.0, cryoSPARC v4.3, MotionCor2, CTFFIND v4.1, Gctf, Topaz v0.2.4.<br>For model building and display: Phenix 1.19.1-4122, COOT 0.9.3 EL, PyMOL v2.5.4, UCSF Chimera v1.15, HOLLOW v1.1.<br>For molecular docking and energy minimization: AutoDock Vina, AutoDock Tools, AMMOS2 web server (http://drugmod.rpbs.univ-paris-diderot.fr/ammosHome.php).<br>For MST binding analysis: MO.Affinity Analysis v2.2.4, Microsoft Excel for Mac 2016.<br>For sequence alignment: ClustalW web server (https://npsa-prabi.ibcp.fr/cgi-bin/npsa_automat.pl?page=/NPSA/npsa_clustalw.html).

For manuscripts utilizing custom algorithms or software that are central to the research but not yet described in published literature, software must be made available to editors and reviewers. We strongly encourage code deposition in a community repository (e.g. GitHub). See the Nature Portfolio guidelines for submitting code & software for further information.

Data

Policy information about availability of data

All manuscripts must include a data availability statement. This statement should provide the following information, where applicable:

- Accession codes, unique identifiers, or web links for publicly available datasets
- A description of any restrictions on data availability
- For clinical datasets or third party data, please ensure that the statement adheres to our policy

The cryo-EM density maps for OaVMAT2TM8/9-BRIL and XlVMAT2WT have been deposited in the Electron Microscopy Data Bank (EMDB) under the accession codes EMD-38389 [https://www.ebi.ac.uk/pdbe/entry/emdb/EMD-38389] (OaVMAT2TM8/9-BRIL); and EMD-38390 [https://www.ebi.ac.uk/pdbe/entry/emdb/EMD-38390] (XlVMAT2WT). The atomic coordinates of the OaVMAT2TM8/9-BRIL and XlVMAT2WT structures have been deposited in the Protein Data Bank (PDB) under the accession codes 8XIT [https://doi.org/10.2210/pdb8XIT/pdb] (OaVMAT2TM8/9-BRIL); and 8XIU [https://doi.org/10.2210/pdb8XIU/pdb] (XlVMAT2WT). Two previously published structures used in this study are available in the Protein Data Bank under accession codes 7BP3 [https://doi.org/10.2210/pdb7BP3/pdb] (hMCT2 dimer) and 8JSW [https://doi.org/10.2210/pdb8JSW/pdb] (hVMAT2-SER). The source data underlying Fig. 1d, 1g, 2c and 2g, Supplementary Fig. 1a, 1c, 1d and 4i, and Supplementary Table 2 and 3 are provided as a Source Data file. Uncropped gels of those presented in Supplementary Figures (1a, 1c, 1d, 1e and 9c) are supplied at the end of the Supplementary Information file.

Research involving human participants, their data, or biological material

Policy information about studies with human participants or human data. See also policy information about sex, gender (identity/presentation), and sexual orientation and race, ethnicity and racism.

Reporting on sex and gender	Human participants or human data are not involved in this research.
Reporting on race, ethnicity, or other socially relevant groupings	Human participants or human data are not involved in this research.
Population characteristics	Human participants or human data are not involved in this research.
Recruitment	Human participants or human data are not involved in this research.
Ethics oversight	Human participants or human data are not involved in this research.

Note that full information on the approval of the study protocol must also be provided in the manuscript.

Field-specific reporting

Please select the one below that is the best fit for your research. If you are not sure, read the appropriate sections before making your selection.

☒ Life sciences ☐ Behavioural & social sciences ☐ Ecological, evolutionary & environmental sciences

For a reference copy of the document with all sections, see nature.com/documents/nr-reporting-summary-flat.pdf

Life sciences study design

All studies must disclose on these points even when the disclosure is negative.

Sample size	The sample size (N=3 biologically independent protein or cell samples) was chosen from similar MST or uptake experiments from former publications, which is a commonly used sample size for MST or uptake assays.
Data exclusions	No data was excluded from analysis.
Replication	All experiments were performed independently for 3 times. All attempts at replication were successful under the experimental conditions defined.
Randomization	Purified protein samples were randomly assigned to different substrate/inhibitor during MST experiments. Cultured HEK293T cells were randomly selected for transfection with various VMAT2 plasmids.
Blinding	Protein samples were purified by multiple researchers. MST measurements and FFN200 uptake assays were performed and analyzed without protein/cell identity information by different researchers.

Reporting for specific materials, systems and methods

We require information from authors about some types of materials, experimental systems and methods used in many studies. Here, indicate whether each material, system or method listed is relevant to your study. If you are not sure if a list item applies to your research, read the appropriate section before selecting a response.

Materials & experimental systems

n/a	Involved in the study
☒	☐ Antibodies
☐	☒ Eukaryotic cell lines
☒	☐ Palaeontology and archaeology
☒	☐ Animals and other organisms
☒	☐ Clinical data
☒	☐ Dual use research of concern
☒	☐ Plants

Methods

n/a	Involved in the study
☒	☐ ChIP-seq
☒	☐ Flow cytometry
☒	☐ MRI-based neuroimaging

Eukaryotic cell lines

Policy information about cell lines and Sex and Gender in Research

Cell line source(s)	The HEK293T cell line (Catalog#: CL-0005) was obtained from Procell (Wuhan, CN).
Authentication	The HEK293T cell line (Catalog#: CL-0005) was authenticated by STR profiling analysis by Procell.
Mycoplasma contamination	The HEK293T cell line (Catalog#: CL-0005) was tested negative for mycoplasma contamination by Procell.
Commonly misidentified lines (See ICLAC register)	No commonly misidentified cell lines were used in this study.

Plants

Seed stocks	Plants are not involved in this research.
Novel plant genotypes	Plants are not involved in this research.
Authentication	Plants are not involved in this research.
